# Commensal Oral Rothia mucilaginosa Produces Enterobactin, a Metal-Chelating Siderophore

**DOI:** 10.1128/mSystems.00161-20

**Published:** 2020-04-28

**Authors:** Carla C. Uranga, Pablo Arroyo, Brendan M. Duggan, William H. Gerwick, Anna Edlund

**Affiliations:** a J. Craig Venter Institute, Genomic Medicine Group, La Jolla, California, USA; b University of California San Diego, Skaggs School of Pharmacy and Pharmaceutical Sciences, La Jolla, California, USA; c Center for Marine Biotechnology and Biomedicine, Scripps Institution of Oceanography, University of California San Diego, La Jolla, California, USA; d University of California San Diego, School of Medicine, Department of Pediatrics, La Jolla, California, USA; University of Southampton

**Keywords:** oral microbiota, *Rothia mucilaginosa*, enterobactin, *Staphylococcus aureus*, *Streptococcus* spp., *Actinomyces timonensis*, *Streptococcus*

## Abstract

Next-generation sequencing studies of saliva and dental plaque from subjects in both healthy and diseased states have identified bacteria belonging to the *Rothia* genus as ubiquitous members of the oral microbiota. To gain a deeper understanding of molecular mechanisms underlying the chemical ecology of this unexplored group, we applied a genome mining approach that targets functionally important biosynthetic gene clusters (BGCs). All 45 genomes that were mined, representing Rothia mucilaginosa, Rothia dentocariosa, and Rothia aeria, harbored a catechol-siderophore-like BGC. To explore siderophore production further, we grew the previously characterized *R. mucilaginosa* ATCC 25296 in liquid cultures, amended with glycerol, which led to the identification of the archetype siderophore enterobactin by using tandem liquid chromatography-mass spectrometry (LC-MS/MS), high-performance liquid chromatography (HPLC), and nuclear magnetic resonance (NMR) spectroscopy. Normally attributed to pathogenic gut bacteria, *R. mucilaginosa* is the first commensal oral bacterium found to produce enterobactin. Cocultivation studies including *R. mucilaginosa* or purified enterobactin revealed that enterobactin reduced growth of certain strains of cariogenic Streptococcus mutans and pathogenic strains of Staphylococcus aureus. Commensal oral bacteria were either unaffected, reduced in growth, or induced to grow adjacent to enterobactin-producing *R. mucilaginosa* or the pure compound. Taken together with *Rothia*’s known capacity to ferment a variety of carbohydrates and amino acids, our findings of enterobactin production add an additional level of explanation to *R. mucilaginosa*’s prevalence in the oral cavity. Enterobactin is the strongest Fe(III) binding siderophore known, and its role in oral health requires further investigation.

**IMPORTANCE** The communication language of the human oral microbiota is vastly underexplored. However, a few studies have shown that specialized small molecules encoded by BGCs have critical roles such as in colonization resistance against pathogens and quorum sensing. Here, by using a genome mining approach in combination with compound screening of growth cultures, we identified that the commensal oral community member *R. mucilaginosa* harbors a catecholate-siderophore BGC, which is responsible for the biosynthesis of enterobactin. The iron-scavenging role of enterobactin is known to have positive effects on the host’s iron pool and negative effects on host immune function; however, its role in oral health remains unexplored. *R. mucilaginosa* was previously identified as an abundant community member in cystic fibrosis, where bacterial iron cycling plays a major role in virulence development. With respect to iron’s broad biological importance, iron-chelating enterobactin may explain *R. mucilaginosa*’s colonization success in both health and disease.

## Abstract (precis)

The communication language of the human oral microbiota is vastly underexplored. However, a few studies have shown that specialized small molecules encoded by BGCs have critical roles such as in colonization resistance against pathogens and quorum sensing. Here, by using a genome mining approach in combination with compound screening of growth cultures, we identified that the commensal oral community member *R. mucilaginosa* harbors a catecholate-siderophore BGC, which is responsible for the biosynthesis of enterobactin. The iron-scavenging role of enterobactin is known to have positive effects on the host’s iron pool and negative effects on host immune function; however, its role in oral health remains unexplored. *R. mucilaginosa* was previously identified as an abundant community member in cystic fibrosis, where bacterial iron cycling plays a major role in virulence development. With respect to iron’s broad biological importance, iron-chelating enterobactin may explain *R. mucilaginosa*’s colonization success in both health and disease.

## INTRODUCTION

Over the past few decades of oral microbiology research, we have come to understand that the oral microbiota is imperative not only for our oral health but also for our overall wellness. Thus far, the oral microbiology research field has focused significant efforts on either describing taxonomic shifts of complex bacterial communities in healthy and disease states or studies of a few model organisms, e.g., cariogenic Streptococcus mutans and the periodontal pathogen Porphyromonas gingivalis, while less attention has been paid to commensal bacteria such as members belonging to the *Rothia* genus. Our knowledge of bacterial taxonomic signatures expands well beyond our knowledge of the functional roles of oral bacteria. However, some studies of small molecules (SMs) (also known as secondary metabolites) secreted by members of the *Streptococcus* genus have illustrated that antimicrobial bacteriocins and nonribosomal peptides play crucial roles in colonization resistance against pathogens (1–3). In addition, recent genome mining studies reveal that the broader oral microbiome is exceptionally rich in biosynthetic gene clusters (BGCs), of which most are unexplored (4–6). Siderophores represent a particularly interesting class of SMs since they not only have the capacity to modulate the human microbiota but also play critical biological roles for the eukaryotic host by serving either as virulence mediators of pathogens or as a stabilizer of the human iron pool (7, 8). Bacteria release siderophores into the surrounding environment, where their apo-form binds metals, resulting in the metal-ligated siderophore, which is imported back into the cell to serve multiple functions in a variety of biochemical mechanisms, such as oxidoreduction of heme-containing proteins (9). Siderophores are also known to reduce oxidative stress deriving from reactive oxygen species (ROS) produced by neighboring bacterial species or human cells (10–12).

Recently, our research team found that the oral microbiome harbors siderophore-like BGCs, of which some belong to the nonribosomal peptide synthetase (NRPS) class (4). Although their structures were not experimentally characterized in this previous study, the NRPS small-molecule products were putatively annotated as griseobactin-like (4). The griseobactin-like BGCs were harbored by oral community members belonging to the ubiquitous and commensal *Rothia* genus, which are previously known for their ability to reduce nitrate to nitrite and thought to limit the growth of aciduric and acidogenic caries pathogens (13, 14). Rothia mucilaginosa is one of the best-studied *Rothia* species and has also been identified as one of the most common community members in healthy subjects compared to subjects with dental caries (15), primary sclerosing cholangitis (a liver and gallbladder disease) (16), oral squamous cell carcinoma (17), and bronchiectasis (18). In contrast to its beneficial associations, recent studies have also shown that *R. mucilaginosa* is associated with dental caries (19, 20) and lung diseases such as pneumonia and cystic fibrosis (CF) (21, 22). In addition, a study of the CF microbiota reported that *R. mucilaginosa* boosts the growth of pathogenic Pseudomonas aeruginosa via cross-feeding mechanisms (14, 22). The functional role of *Rothia* in the human microbiota is not known, but its versatile metabolic capacities, such as fermentation of both complex carbohydrates and peptides/proteins into primary metabolites (e.g., free amino acids, short-chain fatty acids), suggest that one of its ecological roles may be to provide growth substrates for more-specialized community members, such as Gram-negative non-mucus-degrading bacteria (i.e., Pseudomonas aeruginosa [22]). In this study, we explored further the metabolic capacity of *R. mucilaginosa* ATCC 25296 and show that its genome harbors a catechol siderophore BGC that encodes the archetypal siderophore enterobactin, and not griseobactin as we predicted in our previous genome mining study (4). Similar enterobactin-BGCs were identified in all of the genomes of two additional *Rothia* species, R. dentocariosa and R. aeria. Here, we demonstrate that pure enterobactin reduced growth of some cariogenic strains of S. mutans, a few commensal oral *Streptococcus* species, and oral Actinomyces timonensis. It also inhibited formation of the yellow pigment staphyloxanthin and growth of methicillin-resistant strains of Staphylococcus aureus (MRSA). S. aureus is not currently considered a pathogen in oral disease; however, this view is changing as more cases of MRSA and methicillin-susceptible S. aureus (MSSA) have been reported in infections of the oral cavity (23–25). Our study also establishes that enterobactin chelates both zinc and magnesium ions but with less affinity than Fe(III). Conclusively, we describe a new key ecological mechanism that involves the metal-chelating siderophore enterobactin, which not only supports survival and growth of the underexplored yet frequently occurring oral *R. mucilaginosa* but also reduces growth of both cariogenic and multidrug-resistant pathogens.

## RESULTS AND DISCUSSION

More than 2,000 oral bacterial, archaeal, and fungal species have been identified to date, and these are known collectively as the human oral microbiota (26). Some bacterial members of the oral microbiota can cause diseases such as dental caries and periodontal diseases, while others provide colonization resistance against pathogens (27–29). The advent of high-throughput sequencing technologies has greatly increased our understanding of the complexity of the oral microbiota and shed new light not only on the diversity of bacterial taxa but also on their biosynthetic potential, i.e., the widely distributed BGCs that encode small molecules with specialized functions (4–6, 27). Previously, our research team conducted a genome mining survey, targeting BGCs in 461 oral bacterial genomes, which identified a vast unexplored repertoire of ∼5,000 putative BGCs (4). In another study of children with deep dentin caries disease, we found that bacterial community members belonging to the *Rothia* genus, specifically Rothia mucilaginosa, were enriched in saliva from healthy children compared to saliva from children with caries (15). From the same metagenomes, we also identified BGCs that were identical to those harbored by the complete genome sequence representing Rothia mucilaginosa ATCC 25296 (15). Based on this, we employed the oral Rothia mucilaginosa ATCC 25296 as a model species in this study. Here, we expanded on our previous findings and conducted BGC mining of additional *Rothia* genomes and draft genomes (representing *R. mucilaginosa*, *R. dentocariosa*, and *R. aeria*), available in NCBI GenBank, to explore to what extent species within this genus harbor unique BGC signatures, which could explain some of *Rothia*’s competitive success in the oral cavity. By using the antiSMASH 5.0 software (bacterial version) (30), we predicted that each of the 26 Rothia mucilaginosa genomes and draft genomes (available as of 2 February 2020 at https://www.ncbi.nlm.nih.gov/genome/genomes/1812) harbored an NRPS catechol siderophore-like (cat-sid) BGC (see Fig. S1 in the supplemental material). Each of the 12 *R. dentocariosa* draft genomes (available at https://www.ncbi.nlm.nih.gov/genome/genomes/1968) harbored a butyrolactone BGC, a type 1 polyketide synthase (T1PKS) BGC, a lanthipeptide BGC, and a cat-sid BGC. Furthermore, each of the seven *R. aeria* genomes (available at https://www.ncbi.nlm.nih.gov/genome/genomes/12163) harbored a cat-sid BGC and a lasso peptide-like BGC. Taken together, by analyzing genomes representing three different *Rothia* species, unique BGC signatures were observed at the species level and a highly similar NRPS-encoded cat-sid BGC was shared between all three species (Fig. S2), which suggests a broader ecological importance of this siderophore. antiSMASH predicted that the closest homologue to the cat-sid BGC was a mirubactin BGC (14% peptide sequence similarity) (Fig. S1C). Mirubactin is a mixed catecholate and hydroxamate-type NRPS siderophore. However, antiSMASH also predicted that the core building blocks for siderophore biosynthesis were serine and dihydroxybenzoic acid, showing support for the biosynthesis of a catecholate siderophore (Fig. S1).

10.1128/mSystems.00161-20.1 FIG S1 (A and B) Overview of peptide sequence alignments showing the closest homologues to the biosynthetic gene clusters (BGCs) encoding enterobactin produced by Rothia mucilaginosa ATCC 25296 (A) and enterobactin produced by Escherichia coli K-12 (B). Alignments were obtained using the antiSMASH v. 5.0 program (bacterial version). Adenylation domains in both pathways were predicted to select 2,3-dihydroxybenzoic acid (dhb) and serine (ser) as the substrates, respectively. (C) Closeup view of peptide alignment of *R. mucilaginosa* ATCC 25296 BGC and its closest homologue pathways (i.e., mirubactin [14%]; perquinoline A, B, and C [15%]; and steffimycin D [5%]). (D) Closeup view of peptide alignment of E. coli strain K-12 BGC to its closest homologue pathways (i.e., turnerbactin [13%], enterobactin [12%], streptobactin [23%], etc.). The *R. mucilaginosa* BGC could not be aligned with the E. coli BGC due to nonexisting peptide sequence homology in any of the genes except the NRPS genes, which showed 41% homology (Fig. S4). Download FIG S1, TIF file, 2.9 MB. Copyright © 2020 Uranga et al. 2020 Uranga et al. This content is distributed under the terms of the Creative Commons Attribution 4.0 International license.

10.1128/mSystems.00161-20.2 FIG S2 Catecholate siderophore encoding biosynthetic gene clusters identified by the antiSMASH software in genomes of Rothia mucilaginosa ATCC 25296 (I), *R. dentocariosa* M567 (II), and *R. aeria* F0184 (III). Predicted core biosynthetic genes, iron-transporting genes, and genes encoding protein with putative species-specific functions (A to F) are highlighted. Download FIG S2, TIF file, 2.3 MB. Copyright © 2020 Uranga et al. 2020 Uranga et al. This content is distributed under the terms of the Creative Commons Attribution 4.0 International license.

### Screening for the siderophore in Rothia mucilaginosa growth extracts.

**FIG 1:**
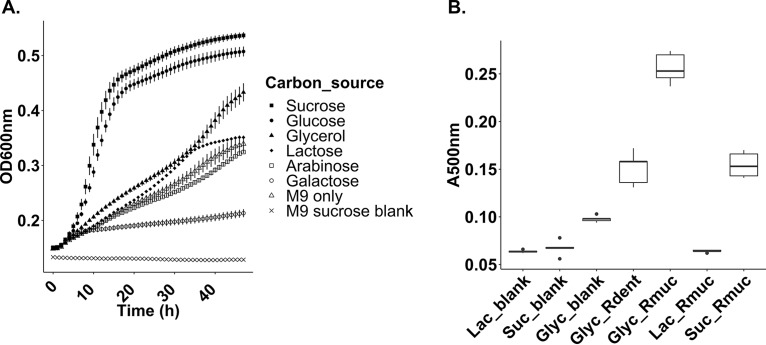
(A) Growth curves for Rothia mucilaginosa ATCC 25296 incubated under aerobic conditions in liquid M9 medium, supplemented with different carbon sources (*x* axis, hours; *y* axis, optical density [OD_600_]). (B) Colorimetric absorbance at 500 nm (*y* axis, *A*_500_) capturing catecholate derivatives in liquid *R. mucilaginosa* (Rmuc) and *R. dentocariosa* M567 (Rdent) growth cultures (*x* axis) using Arnow’s assay. *R. mucilaginosa* was grown in M9 medium supplemented with 100 mM glycerol (Glyc_Rmuc), 100 mM lactate (Lac_Rmuc), and 100 mM sucrose (Suc_Rmuc). *R. dentocariosa* was grown in 100 mM glycerol to see if glycerol induced siderophore production as seen for *R. mucilaginosa* cultures.

To test if Rothia mucilaginosa ATCC 25296 secreted a siderophore while growing in liquid growth cultures, we tested growth under multiple conditions. We specifically optimized for growth in minimal medium to reduce the number of metabolites that could interfere with our downstream mass spectrometric analysis and purification of the siderophore. Plateauing growth curves of *R. mucilaginosa* ATCC 25296 were obtained in liquid minimal medium M9 cultures supplemented with either 100 mM sucrose or glucose during aerobic incubation at 37°C (Fig. 1A). However, when incubated with other carbon sources (glycerol, lactose, arabinose, or galactose), growth was reduced (Fig. 1A). To explore if a siderophore was produced under any of the above culture conditions, we screened liquid growth extracts using two different assays: a hydroxamate assay that targets carboxylate siderophores (31) and Arnow’s assay, which targets catecholate siderophores (32). Arnow’s assay showed clear colorimetric changes as the normally colorless M9 medium turned ruby red in cultures incubated with sucrose and glycerol, while the hydroxamate assay showed no color change. The presence of a catecholate siderophore in these cultures was also confirmed using absorbance measurements at 500 nm, which are known to capture catecholate derivatives (33). Not only did these results reveal that *R. mucilaginosa* ATCC 25296 can produce a catechol siderophore, but they also demonstrated that the antiSMASH program could accurately predict the correct core building blocks (serine and dihydroxybenzoic acid). To facilitate compound isolation and purification, we further explored if glycerol, which is known to elicit secondary metabolite production in other actinobacteria (34), could increase siderophore yields. This was indeed the case as enterobactin production increased significantly in the glycerol-amended cultures (absorbance at 500 nm, ∼0.25) (Fig. 1B). However, under the same conditions, growth decreased (Fig. 1A). We also tested if liquid cultures of Rothia dentocariosa M567, which also harbors a cat-sid BGC (Fig. S2), can produce a siderophore when subjected to glycerol cultivation (Fig. 1B). Production was observed for this species as well (absorbance at 500 nm, ∼0.15) but not to the same extent as for *R. mucilaginosa* (Fig. 1B).

### Detailed characterization of *R. mucilaginosa*’s catechol siderophore and cat-sid BGC.

Using Phyre2, a protein structure prediction software program (35), only one gene within the enterobactin BGC from *R. mucilaginosa* ATCC 25296 showed amino acid sequence homology to the BGC that encodes enterobactin in the Escherichia coli JM109 genome (i.e., amino acid location 51 to 463 in the NRPS gene [Fig. S3]). The overall low structural BGC homology as well as the low amino acid sequence homology between the NRPS genes in *R. mucilaginosa* and E. coli BGCs illustrates that the local gene environments surrounding the core biosynthetic NRPS genes have diversified greatly between different taxonomic groups of bacteria and have no influence on the final compound structure.

10.1128/mSystems.00161-20.3 FIG S3 Results from peptide sequence alignment analysis of the NRPS gene in the Rothia mucilaginosa ATCC 25296 cat-sid BGC using the Phyre2 protein structure prediction tool showed 41% sequence homology to the EntE/EntB fusion protein harbored by the enterobactin BGC from Escherichia coli JM109. Download FIG S3, TIF file, 2.5 MB. Copyright © 2020 Uranga et al. 2020 Uranga et al. This content is distributed under the terms of the Creative Commons Attribution 4.0 International license.

**FIG 2:**
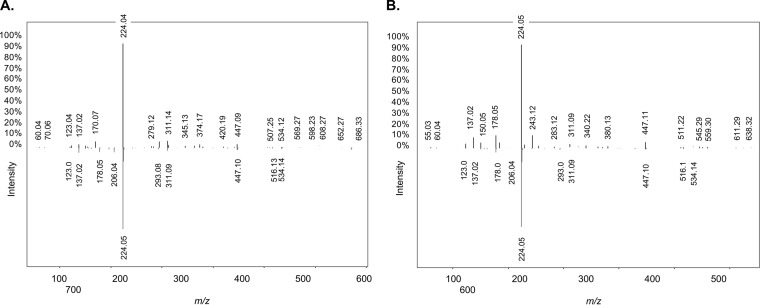
Replicate mirror mass fragmentation patterns for enterobactin (*m/z* 670.152) produced by Rothia mucilaginosa ATCC 25296 (ion fragments pointing up) and enterobactin from the gold standard spectrum (ion fragments pointing down) in the Global Natural Products Social Molecular Networking (GNPS) library (36). Six major fragments (*m/z* 123.04, *m/z* 137.02, *m/z* 224.05, *m/z* 311.09 to 311.14, *m/z* 447.09 to 447.11, and *m/z* 534.14) of the query compound matched the gold standard in GNPS. (A) Purified extract derived from *R. mucilaginosa* ATCC 25296 growth medium (negative ionization mode). (B) Further enrichment of the siderophore using thin-layer chromatography (positive ionization mode). Both experiments confirmed the production of enterobactin.

**FIG 3:**
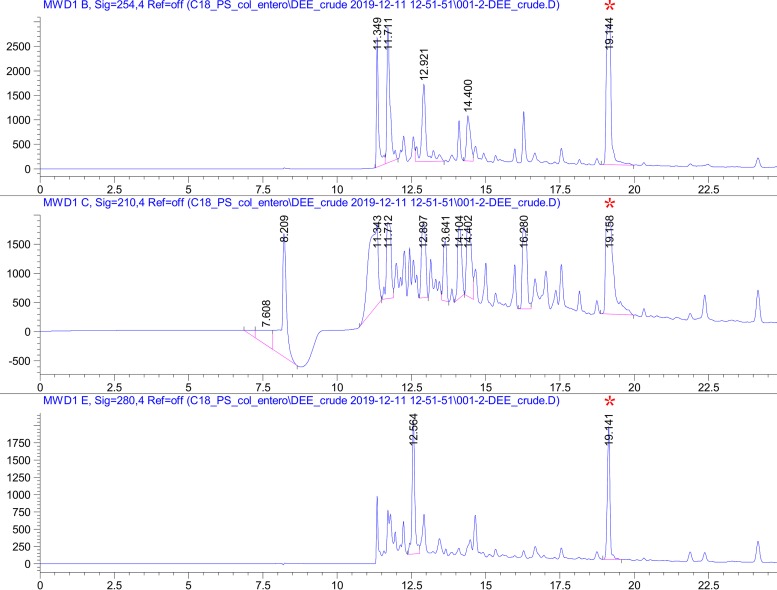
High-performance liquid chromatography (HPLC) purification traces of enterobactin from Rothia mucilaginosa ATCC 25296 crude growth extract, measured at 210 nm, 254 nm, and 280 nm using a solvent gradient from 30 to 65% buffer B. The peak at 19.141 min (red asterisk) was eluted and further analyzed by NMR. *x* axis, minutes; *y* axis, peak absorbance intensity.

Preliminary high-resolution mass spectrometry (liquid chromatography-tandem mass spectrometry [LC-MS/MS]) analysis of an *R. mucilaginosa* ATCC 25296 siderophore-enriched sample (derived from a glycerol-amended growth culture) in negative ionization mode yielded a compound with a parent mass [M+H] of 670.152 Da. Upon collision-induced dissociation (CID) fragmentation of the parent mass, and comparative analysis with all previously characterized compound spectra available through the Global Natural Products Social Molecular Networking (GNPS) library (36), it became clear that the ion fragments matched the well-characterized catecholate siderophore enterobactin (*m/z* 670.164, GNPS gold standard spectrum CCMSLIB00005435752, GNPS cosine score of 0.71 to 0.78) (Fig. 2A). To verify our findings, we performed additional LC-MS/MS on purified extracts from thin-layer chromatography, which again resulted in the identification of enterobactin or a close homologue using the GNPS infrastructure, this time in positive ionization mode (Fig. 2B). Further purification of this compound using high-performance liquid chromatography (HPLC) showed a well-separated peak that tested positive in the Arnow assay (Fig. 3). This peak was eluted and analyzed by ^1^H nuclear magnetic resonance (NMR) to confirm that *R. mucilaginosa* ATCC 25296 produces enterobactin (Table S1). All observed chemical shift values in Table S1 (obtained from *R. mucilaginosa*’s enterobactin) were also reported previously in an infrared (IR) and NMR spectroscopic study of the archetypal enterobactin molecule (37). Proton and two-dimensional (2D) NMR spectra (heteronuclear single quantum correlation [HSQC], heteronuclear multiple-bond coherence [HMBC], and proton correlation spectroscopy [COSY]) for the *R. mucilaginosa*-derived compound confirmed its identity (Fig. S4).

10.1128/mSystems.00161-20.4 FIG S4 (A) ^1^H NMR spectrum of enterobactin purified from *R. mucilaginosa* ATCC 25296. (B) 2D heteronuclear single quantum correlation (HSQC) NMR spectrum of enterobactin purified from *R. mucilaginosa* ATCC 25296. (C) 2D heteronuclear multiple bond coherence (HMBC) NMR spectrum of enterobactin purified from *R. mucilaginosa* ATCC 25296. (D) 2D proton correlation spectroscopy (H COSY) NMR spectrum of enterobactin purified from *R. mucilaginosa* ATCC 25296. Download FIG S4, TIF file, 2.8 MB. Copyright © 2020 Uranga et al. 2020 Uranga et al. This content is distributed under the terms of the Creative Commons Attribution 4.0 International license.

10.1128/mSystems.00161-20.8 TABLE S1 Experimental ^13^C and ^1^H chemical shifts (ppm) of enterobactin produced by Rothia mucilaginosa ATCC 25296 in DMSO-d_6_. All chemical shifts in this work are identical to the already-characterized enterobactin compound (37). Download Table S1, PDF file, 0.03 MB. Copyright © 2020 Uranga et al. 2020 Uranga et al. This content is distributed under the terms of the Creative Commons Attribution 4.0 International license.

### Enterobactin activity screening using cocultivation assays.

**FIG 4:**
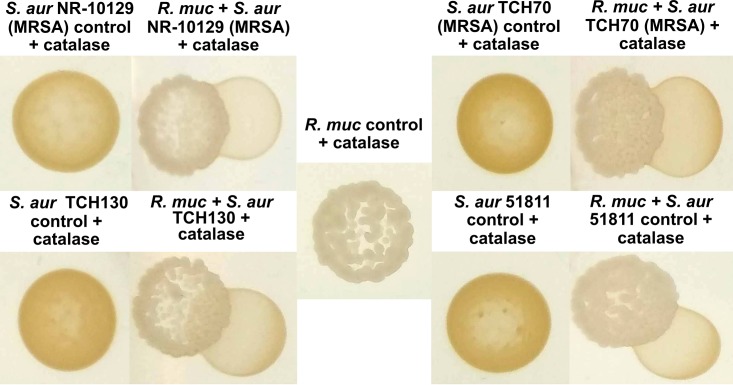
Rothia mucilaginosa ATCC 25296 (*R. muc*) inhibits pigment production in Staphylococcus aureus NR-10129 (MRSA), S. aureus TCH70 (MRSA), S. aureus TCH130, and enterotoxin H-producing S. aureus ATCC 51811, on M9 agar plates (100 mM glycerol) with 8 μg/ml catalase added. All S. aureus strains show yellow pigmentation when growing alone.

**FIG 5:**
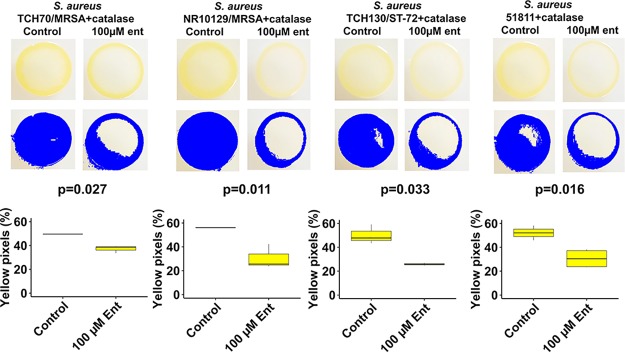
Yellow pigmentation of S. aureus strains exposed to 100 μM enterobactin and 8 μg/ml catalase on M9 agar plates supplemented with 100 μM glucose. Yellow pigmentation was measured with the R package “countcolors” (51). All strains presented statistically significant reductions in pigmentation in the presence of 100 μM enterobactin purified from *R. mucilaginosa* ATCC 25296 and catalase (*P* < 0.05, two-tailed *t* test). Box plots from yellow pixel measurements were generated with the R Studio program (49) and ggplot2 (50).

**FIG 6:**
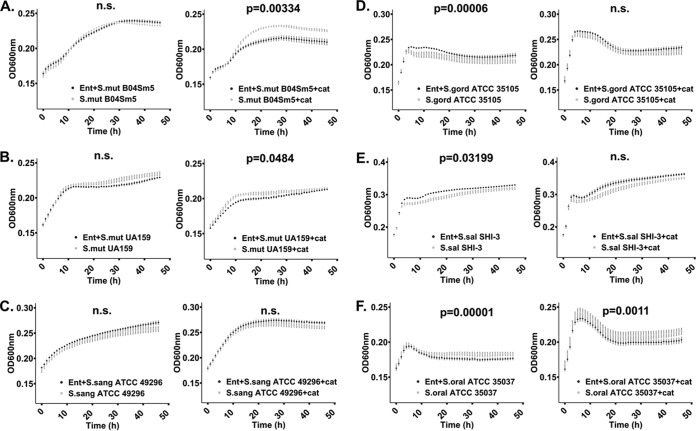
Growth curves of cariogenic and commensal *Streptococcus* species. Bacteria were grown aerobically at 37°C in liquid M9 medium supplemented with 100 mM glucose, 1 μM FeCl_3_, either with or without 100 μM enterobactin purified from *R. mucilaginosa*, and with or without 8 μg/ml catalase. Growth curves in black represent cultures amended with enterobactin. Statistically significant growth curves (*P* < 0.05) are shown with corresponding *P* values. Error bars reflect the standard error of the mean (calculated from triplicates). n.s., not significant. (A) Growth of cariogenic S. mutans strain B04Sm5 with enterobactin only (left panel) and with enterobactin and catalase (right panel). (B) Growth of cariogenic S. mutans strain UA159 with enterobactin only (left panel) and with enterobactin and catalase (right panel). (C) Growth of commensal S. sanguinis ATCC 49296 with enterobactin only (left panel) and with enterobactin and catalase (right panel). (D) Growth of S. gordonii ATCC 35101 with enterobactin only (left panel) and with enterobactin and catalase (right panel). (E) *S. salivarius* strain SHI-3 with enterobactin only (left panel) and with enterobactin and catalase (right panel). (F) Growth of S. oralis ATCC 35037 with enterobactin only (left panel) and with enterobactin and catalase (right panel). Graphs were generated and statistically validated using R Studio and the “statmod” and “ggplot2” packages (48–50).

Interactions between *R. mucilaginosa* ATCC 25296 or the purified enterobactin compound and other bacterial species were studied by employing cocultivation agar assays and liquid growth assays. On agar plates, growth of *R. mucilaginosa* was established prior to spotting the challenging species. Interactions were initially studied on both rich (brain heart infusion [BHI]) and minimal M9 agar media to investigate under which conditions *R. mucilaginosa* could modulate growth of the competitor test strain, potentially via iron competition (visualized as clearing zones surrounding *R. mucilaginosa*). In the liquid monocultures, the purified siderophore was added at the same time as the cultures were seeded with the test strain. We found that while growing on minimal M9 agar, supplemented with sucrose (100 mM) during plate interaction studies, *R. mucilaginosa* inhibited growth of Actinomyces timonensis DSM 23838 (Fig. S5A) and reduced pigment production in four strains of pathogenic Staphylococcus aureus in the presence of catalase (Fig. 4 and Fig. S6). We added catalase to the agar plates to prevent growth inhibition by eventual reactive oxygen species (ROS) produced by *R. mucilaginosa*. The results suggest that inhibition of pigment production is not due to ROS but actually due to enterobactin. To further elucidate this interaction, we conducted monococulture experiments on agar plates where we added the purified enterobactin to S. aureus agar plates (Fig. 5). With catalase added, statistically significant differences in pigmentation were observed for all S. aureus strains amended with enterobactin, including MRSA strains TCH70/MRSA and NR10129 (*P* < 0.05, two-tailed *t* test) (Fig. 5), confirming an important role of enterobactin in the inhibition of S. aureus virulence and growth (38, 39). For all the tested oral commensal and pathogenic *Streptococcus* species, no growth inhibition was observed on agar plates. However, changes in growth were confirmed for some of the species when adding the purified enterobactin compound to liquid cultures (with or without catalase) (Fig. 6). Of the pathogenic *Streptococcus* strains tested in liquid growth cultures amended with enterobactin and catalase, we observed that growth of both S. mutans UA159 and S. mutans B04Sm5 was significantly reduced (*P* < 0.05, *t* test of averaged mean values) (Fig. 6A and B), elucidating that *R. mucilaginosa* has the capacity to reduce growth of cariogenic pathogens, via iron competition. Taken together, these results show that growth and virulence responses to enterobactin are varied for strains belonging to the same species, which warrants further exploration in future studies. The commensal oral bacteria that were tested showed various responses to enterobactin and catalase (for a list of species tested, see Materials and Methods). For example, growth of Streptococcus sanguinis ATCC 49296 was not significantly impacted by enterobactin (Fig. 6C). However, when subjected to catalase treatment (with and without enterobactin), the S. sanguinis growth rate increased as optical density at 600 nm (OD_600_) reached 0.25 after 10 h of growth compared to the non-catalase-amended cultures (an OD of 0.25 was reached after 20 h) (Fig. 6C). This reflects that catalase protects S. sanguinis from oxidative damage caused by its own ROS production and thereby stimulates its growth (40). Growth of Streptococcus gordonii ATCC 35105 was significantly enriched by enterobactin but only without catalase (*P* < 0.0001, *t* test of averaged mean values), which illustrates that growth was boosted by the presence of enterobactin (Fig. 6D). A similar trend was observed for Streptococcus salivarius SHI-3, which also increased significantly in cultures amended with enterobactin when no catalase was added (*P* < 0.05, *t* test of averaged mean values) (Fig. 6E). Conversely, Streptococcus oralis grew significantly more in the presence of catalase only (*P* < 0.0001, *t* test of averaged mean values), while growth was reduced by enterobactin (*P* < 0.05, *t* test of averaged mean values) (Fig. 6F). These results show that bacteria belonging to the *Streptococcus* genus have a varied response to enterobactin in the presence or absence of ROS. The liquid culture experiments demonstrate that *S. salivarius* and S. gordonii can benefit from the presence of enterobactin-producing *R. mucilaginosa* while S. oralis growth can be reduced. A growth boost of *S. salivarius* was also observed on coculture agar plates (minimal M9 medium supplemented with sucrose) adjacent to *R. mucilaginosa* (Fig. S5B). Whether this response was due to the presence of enterobactin needs to be explored further.

10.1128/mSystems.00161-20.5 FIG S5 (A) Rothia mucilaginosa ATCC 25296 growth was established first on M9 agar (100 mM sucrose) (required for growth of the challenging species Actinomyces timonensis DSM 23838 under aerobic conditions). *A. timonensis* was spotted adjacent to *R. mucilaginosa*, and its growth was inhibited. (B) Streptococcus salivarius SHI-3 presents a growth boost and forms growth on top of *R. mucilaginosa* when plated adjacent to *R. mucilaginosa* on M9 minimal agar medium (100 mM sucrose). Download FIG S5, TIFF file, 2.9 MB. Copyright © 2020 Uranga et al. 2020 Uranga et al. This content is distributed under the terms of the Creative Commons Attribution 4.0 International license.

10.1128/mSystems.00161-20.6 FIG S6 Rothia mucilaginosa ATCC 25296 inhibits pigment production in Staphylococcus aureus enterotoxin H-producing strain ATCC 51811 and MRSA strain TCH70 growing on M9 agar plates with no catalase added (100 mM glycerol). Download FIG S6, TIF file, 2.8 MB. Copyright © 2020 Uranga et al. 2020 Uranga et al. This content is distributed under the terms of the Creative Commons Attribution 4.0 International license.

**FIG 7:**
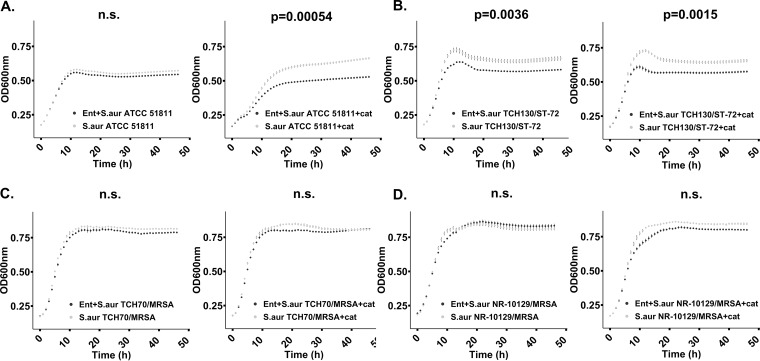
Reduced growth of Staphylococcus aureus incubated in liquid M9 growth medium (100 μM enterobactin, 8 μg/ml catalase, 100 mM glucose) at 37°C for 24 h. No enterobactin was added to the control samples. Growth curves in black represent cultures amended with enterobactin. Statistically significant growth curves (*P* < 0.05) are shown with corresponding *P* values. Error bars reflect the standard error of the mean (calculated from triplicates). (A) Growth of S. aureus strain 51811 with enterobactin only (left panel) and with enterobactin and catalase (right panel). (B) Growth of S. aureus strain TCH130/ST-72 with enterobactin only (left panel) and with enterobactin and catalase (right panel). (C) Growth of S. aureus TCH70/MRSA with enterobactin only (left panel) and with enterobactin and catalase (right panel). (D) Growth of S. aureus NR-10129/MRSA with enterobactin only (left panel) and with enterobactin and catalase (right panel). Graphs were generated and statistically validated using R Studio and the “statmod” and “ggplot2” packages (48–50).

Liquid growth culture experiments including enterobactin were also performed for pathogenic Staphylococcus aureus strains. Growth of the enterotoxin H-producing strain S. aureus ATCC 51811 and the MRSA strain S. aureus TCH130 130/ST-72 was significantly reduced by enterobactin when catalase was present, which suggests that enterobactin negatively impacts S. aureus growth (*P* < 0.005, *t* test of averaged mean values) (Fig. 7A and B). None of the other tested MRSA strains showed reduced growth in enterobactin-amended cultures (with or without catalase added) (Fig. 7C and D). Interestingly, our findings demonstrate that strains belonging to the same bacterial species show different responses to enterobactin, which warrant further investigations of the role of strain-level sensitivity to this compound in MRSA virulence and growth.

To evaluate if enterobactin was actively chelating free iron throughout the liquid cultivation experiments, we incubated 500 μM purified compound in sterile M9 liquid medium as described for bacterial liquid cultures. By using Arnow’s assay, we observed that its binding capacity to molybdenum (which in Arnow’s assay substitutes for iron) was reduced by 14% after 6 h of incubation. Assuming that this response is linear, we estimated that 56% of enterobactin’s activity was lost after 24 h. This suggests that the compound became inactive in our coculture experiments over time for physiochemical reasons and that a more dramatic effect of enterobactin likely would have been observed if its activity had remained stable.

We also investigated if enterobactin can bind other metal ions by using the calmagite assay, which is normally used for testing the hardness of water by detecting ions such as magnesium (Mg), calcium (Ca), and zinc (Zn) that bind to EDTA (41, 42). EDTA is used as a reference compound due to its well-known chelation of divalent cations. Results from this test revealed that enterobactin can chelate both Mg^2+^ and Zn^2+^ ions. Enterobactin showed a chelation activity of 40 μM EDTA-equivalents at a concentration of 100 μM, which is 2.5 times less than EDTA affinity for Mg^2+^ and Zn^2+^ (Fig. S7). These results establish that enterobactin can indeed chelate additional ions and not only ferric iron (Fe^3+^), which could have critical impacts on other microbial community members as well as the human host. It is well known that Zn^2+^ is a critical component of the ubiquitous protein zinc fingers that are able to interact directly with DNA, RNA, and proteins. Magnesium (Mg^2+^) is involved in nervous system signaling, immune system function, and bone formation. Our findings show that *R. mucilaginosa* has the potential to compete with the host not only for Fe^3+^ but also for Mg^2+^ and Zn^2+^, which suggest that *R. mucilaginosa* could play an important role in health and disease outcomes.

10.1128/mSystems.00161-20.7 FIG S7 Standard curves for the calmagite compleximetric assay for siderophore activity. (A) A 25 μM concentration of MgSO_4_ complexed with calmagite. (B) A 25 μM concentration of ZnSO_4_ complexed with calmagite. Both curves arise from dilutions of EDTA from 0 to 500 μM added to the calmagite-metal complex at pH 10 and a color change from red to blue monitored at 650 nm (41, 42). Enterobactin at 100 μM bound metal ions equal to the amount bound by 40 μM EDTA. Download FIG S7, TIF file, 2.9 MB. Copyright © 2020 Uranga et al. 2020 Uranga et al. This content is distributed under the terms of the Creative Commons Attribution 4.0 International license.

Our study reveals that *R. mucilaginosa* ATCC 25296 produces the archetype siderophore enterobactin when growth conditions are suboptimal in a minimal growth medium supplemented with glycerol, a known inducer of secondary metabolites in *Streptomyces* (43). We observed different growth responses to purified enterobactin by various members of the oral microbiota: some commensal *Streptococcus* species increased in growth while others decreased (independently in the presence of ROS). Our findings also demonstrate that growth of pathogenic bacteria (i.e., two cariogenic strains of S. mutans and methicillin-resistant strains of S. aureus) is reduced in growth in cultures amended with enterobactin. Moreover, while methicillin-resistant S. aureus grew adjacent to enterobactin-producing *R. mucilaginosa* ATCC 25296 (with or without ROS present), the virulence factor known as golden pigmentation (staphyloxanthin) was highly reduced. While we provide evidence that the loss of this golden pigmentation is caused by enterobactin, the molecular mechanisms of the interaction remain to be explored. Further examination of the role of *R. mucilaginosa* enterobactin in inhibiting pigment production and bacterial growth may provide a pathway toward the development of new therapeutic leads against not only MRSA strains but also other pathogens, such as cariogenic Streptococcus mutans.

In conclusion, the role of enterobactin in health and dysbiosis of the oral microbiota is yet unexplored. However, based on previous research findings of enterobactin’s role in various pathogenicities, it is difficult to overestimate the significance of its iron-chelating capacity. It is possible that enterobactin-producing *R. mucilaginosa* can supply pathogens with additional iron by a siderophore-sharing mechanism and thereby fuel virulence. The fact that bacteria belonging to the *Rothia* genus not only represent one of the most prevalent oral bacterial groups but also have the capacity to control the availability of iron, both for the host and for other oral microbial community members, illustrates how critical it is to explore the ecological and clinical roles of enterobactin production by *Rothia* further.

## MATERIALS AND METHODS

### Bacterial isolates used in this study.

The isolates used in this study included Rothia mucilaginosa ATCC 25296, Rothia dentocariosa M567, Streptococcus mitis ATCC 6249, Streptococcus salivarius SHI-3 (isolate from oral *in vitro* biofilms) (44), Streptococcus sanguinis ATCC 49296, Streptococcus oralis ATCC 35037, Streptococcus gordonii ATCC 35105, Actinomyces timonensis DSM 23838, Streptococcus mutans UA159, Streptococcus mutans B04Sm5, Staphylococcus aureus strain MN8 (MSSA), Staphylococcus aureus strain NR-10129 (MRSA), Staphylococcus aureus strain TCH130/ST-72, Staphylococcus aureus ATCC 51811 (enterotoxin H producer), and Staphylococcus aureus TCH70 (MRSA).

### Growth media used in this study.

A minimal medium was modified and developed from the M9 medium recipe reported by Elbing and Brent (45). All glassware was rinsed thoroughly with 2.5 M HCl and washed with deionized (DI) H_2_O before use. The fundamental M9 medium formulation (pH 7) was supplemented with 0.8% deferrated acid-hydrolyzed Casamino Acids (BD Biosciences, San Jose, CA, USA), 8 mM MgSO_4_ (Millipore-Sigma, Carlsbad, CA, USA), and various carbon sources (sucrose, glycerol, galactose, lactose, arabinose, glucose, or lactate), to a final concentration of 100 mM. The acid-hydrolyzed Casamino Acids were deferrated with an equal volume of 3% 8-hydroxyquinoline (Fisher Chemical, Pittsburgh, PA, USA) in chloroform. MgSO_4_, glucose, and sucrose were added postautoclaving to prevent solution clouding and the Maillard reaction (“caramelization” of sugars with amino acids). Plates were made of the same formulations, containing 1% agar. Nutrient- and iron-rich brain heart infusion (BHI) media (Oxoid, Thermo Scientific, Carlsbad, CA, USA) were also used in liquid cultures and agar plates for cultivating experimental strains.

### Mining for BGCs in *Rothia* genomes.

The biosynthetic gene cluster (BGC) prediction program antiSMASH, bacterial version (30), which is able to predict core secondary metabolite structures from BGC sequences, was used to identify BGCs in 26 genomes (including both completed and draft genomes) representing *R. mucilaginosa*, available at https://www.ncbi.nlm.nih.gov/genome/genomes/1812?; 12 genomes (including both completed and draft genomes) representing *R. dentocariosa*, available at https://www.ncbi.nlm.nih.gov/genome/genomes/1968?; and 7 genomes (one full-length and four draft genomes) representing *R. aeria*, available at https://www.ncbi.nlm.nih.gov/genome/genomes/12163?. The NaPDoS program was used for further classification of the nonribosomal peptide synthase (NRPS) catechol siderophore BGCs by using the C-domain classification tool (46).

### BGC structural analysis.

For structural homology modeling and comparative analysis of the NRPS protein harbored by the Rothia mucilaginosa ATCC 25296 cat-sid BGC, Phyre2 software (35) was used to compare the NRPS with known structures in the Phyre2 database.

### Cocultivation agar assays.

For bacterial interaction screening studies, all bacterial isolates were seeded from glycerol stocks in BHI (Oxoid, Thermo Scientific) medium and incubated for 24 h before placement on either BHI agar or M9 minimal agar medium supplemented with either 100 mM sucrose (Acros Organics, Pittsburgh, PA, USA), glucose (Millipore-Sigma), or glycerol (Honeywell, Mexico City, Mexico). Plates were incubated either in a 5% CO_2_ incubator at 37°C or anaerobically at 37°C in an anaerobic chamber (Coy Laboratory Products, Grass Lake, MI, USA) with a gas mix of 5% H_2_, 5% CO_2_, and 90% N_2_. All tested strains were grown similarly except *A. timonensis*, which was incubated for 48 h in BHI in an anaerobic chamber at 37°C before plating due to its lower growth rate in M9 minimal medium. *R. mucilaginosa* or *R. dentocariosa* was plated by dropping 20 to 30 μl, in three to six replicates, onto plates, allowing the drops to air dry before incubation. Control incubations consisted of 20- to 30-μl drops of each tested bacterial species. Agar plates with bacteria were incubated 24 to 48 h prior to adding another species. Interactions with other bacteria were tested by adding a 20- to 30-μl drop next to the first strain, making sure the drop made contact with the established bacterial strain. Interactions were assessed after another 24 to 76 h of incubation. Interactions were screened with the naked eye for growth inhibitions zones or other growth-related behaviors. All interaction assays were repeated at least three times and documented.

### Siderophore detection assays.

To clarify the type of siderophore produced by *R. mucilaginosa* ATCC 25296 and *R. dentocariosa* M567, siderophore-positive supernatants were assayed using Arnow’s assay (32) for catecholate compounds, and the iron perchlorate assay for hydroxamate siderophores (31). For the hydroxamate siderophore assay, 1.0 ml of a 10 mM solution of Fe(CIO_4_)_2_ (Alfa Aesar, Tewksbury, MA, USA) in 0.1 M HClO_4_ (Ricca Chemical, Visalia, CA, USA) was mixed with 1.0 ml of unknown and the absorbance was read at 495 nm. For Arnow’s assay, cultures were screened by mixing 200 μl culture with 20 μl 5 M HCl (Ricca Chemical), followed by 100 μl Arnow’s reagent (20% sodium molybdate dihydrate [EMD, St. Louis, MO, USA] and 20% sodium nitrite [Fisher Chemical]) in DI H_2_O. To develop the ruby red color indicating the presence of a catecholate siderophore, 20 μl 10 N NaOH (Ricca Chemical) was added. These were assessed visually for culture and HPLC fraction screening. For quantitative assays, these were measured spectrophotometrically at 500 nm.

### Siderophore purification and structural elucidation.

Siderophore enrichment in growth cultures of *R. mucilaginosa* ATCC 25296 was implemented in several steps. First, after 7 days of incubation, the supernatant of a liquid culture was acidified to pH 2. Twenty grams per 100 ml of Amberlite XAD16 (Alfa Aesar) was added to the supernatant, which was then placed in an orbital shaker at 175 rpm and shaken overnight. The Amberlite was collected, washed three times with DI H_2_O, and then extracted with diethyl ether (DEE) (Millipore-Sigma) and methanol (Millipore-Sigma). The solvent phase (the top diethyl ether layer) was evaporated at room temperature in an open container. The material was separated by silica gel thin-layer chromatography (TLC) (Millipore-Sigma); developed in a mixture of 65% *n*-butanol (Acros Organics), 25% acetic acid (Fisher Scientific, Pittsburgh, PA, USA), and 15% water; and then developed with Arnow’s reagent. The Arnow-positive TLC band was removed and reextracted with DEE, solvent was evaporated, and the remaining material was tested with Arnow’s assay. Mass spectrometry (LC-MS/MS) using a triple time of flight (TOF) mass spectrometer (AB Sciex 5600; Framingham, MA, USA) was done in both positive and negative modes on a PS C_18_ column (Phenomenex, Torrance, CA; 2.6-μm particle size, 4.6-mm diameter, 250-mm length) and eluted with a gradient of 0 to 100% acetonitrile and 0.1% formic acid (Honeywell, Mexico City, Mexico). Upon obtaining data, the msConvert program was used to convert vendor files to the mzXML format (47). The Global Natural Products Social Molecular Networking (GNPS) platform was used to putatively annotate the detected fragment masses (36). For siderophore purification, the crude DEE extract was resuspended in 50% methanol-water and purified with the PS C_18_ column using a 20-min gradient of 30% to 65% buffer B (acetonitrile with 0.1% formic acid) using an Agilent 1200 series HPLC with a fraction collector. Buffer A consisted of water with 0.1% formic acid. The pure HPLC fraction was diluted with DI H_2_O, frozen at −80°C, and lyophilized to remove solvents and water. For NMR, pure lyophilized enterobactin was resuspended in dimethyl sulfoxide (DMSO)-d_6_ and analyzed via nuclear magnetic resonance (NMR) with a 600-MHz (14.1-T) Bruker Avance III NMR fitted with a 1.7-mm inverse detection triple resonance cryoprobe with z-gradients.

### Liquid cocultivation assay with pure enterobactin.

In order to gain further insight into the effects of enterobactin on bacterial growth, liquid growth experiments were prepared with purified enterobactin from *R. mucilaginosa*. Because hydrogen peroxide can also be responsible for growth inhibition, catalase (also known as hydrogen peroxide oxidoreductase) was used in the culture medium to degrade hydrogen peroxide and isolate the effect of the siderophore itself. Briefly, to enrich for growth of each bacterial species that was included in cocultivation experiments in minimal M9 medium, cultures were first established in BHI from frozen glycerol stocks and incubated for 24 h aerobically at 37°C with 5% CO_2_. For the growth curve assays, glucose was chosen as a universal carbon source to accommodate all strains used, some of which are not able to grow on glycerol. BHI medium was removed from the cells prior to starting experiments in minimal M9 medium as follows: 1 ml of each culture was centrifuged at 7,000 rpm for 10 min, the supernatant was removed from the pellet using a sterile pipette, and the pellet was resuspended in an assay medium consisting of 0.5 ml minimal M9 medium supplemented with 100 mM glucose, 8 mM MgSO_4_, 0.8% deferrated amino acids, and 1 μM FeCl_3_, either with or without 8 μg/ml catalase (Millipore-Sigma). The bacterial cell suspensions were further diluted 1:20 in the same medium. A stock solution of enterobactin was prepared for the assay by suspending purified enterobactin in M9 plus 100 mM glucose, 8 mM MgSO_4_, and 0.8% deferrated amino acids with or without catalase to 200 μM. One hundred microliters of the 200 μM siderophore suspension was added to a 96-well plate, in triplicate, for each bacterial strain studied. One hundred microliters of the 1:20 bacterial suspension was added to each well, in triplicate for each condition. The control consisted of the same preparations, but without the siderophore. All plates were incubated at 37°C for 48 h in a Tecan Infinite M Nano spectrophotometer, and growth was monitored every hour by absorbance measurements at 600 nm. Statistical analysis was done with the R package “statmod” using the “compareTwoGrowthCurves” function with an nsim parameter value of 100,000 (48) based on a mean T calculation. All graphical figures were generated using R Studio, version 1.2.5001 (49), and the package ggplot2 (50).

### Agar plate assays of S. aureus strains amended with pure enterobactin from *R. mucilaginosa*.

To assess growth and staphyloxanthin pigmentation (also known as golden pigment) production of S. aureus strains on M9 agar plates supplemented with glucose and pure enterobactin, 24-h cultures of S. aureus strains grown in BHI were premixed 1:2 with a 2× solution containing 2× M9 medium, 200 mM glucose, 16 μg/ml catalase, and 200 μM enterobactin so that final concentrations were 1× M9, 100 mM glucose, 8 μg/ml catalase, and 100 μM enterobactin. Controls consisted of the exact same formulation but without enterobactin. Twenty-microliter drops were plated onto M9 agar plates (100 mM glucose) as three replicates and incubated 24 h at 37°C and 5% CO_2_. The R package “countcolors,” version 0.9.1 (51), was used for quantifying the level of yellow pigmentation in the bacterial growth areas by setting a pixel color detection range using only the red-green-blue (RGB) values detected in the images obtained. A rectangular range of color (RGB scale) values for yellow pigmentation detection used were upper RGB values of 0.988235294, 0.960784314, and 0.823529412 and lower RGB values of 0.91372549, 0.850980392, and 0.396078431. The fractions of yellow pixels per image were quantified in this way and converted to percent values, indicating the percentage of yellow pixels detected in each image. The command in the “countcolors” package used was “countcolors::countColorsInDirectory,” in order to automate yellow pigmentation parameters for all images in the image folder and to standardize detection across the strains and replicates. Separate images were created with the results in order to reveal the detection of yellow pixels by the software. These separate images show the pixels that were detected and counted by the R program “countcolors.” The yellow pixels were automatically replaced with blue by the program to better visualize the effect of enterobactin on S. aureus pigmentation. Three images per condition and strain were measured for yellow pigmentation, and a two-tailed *t* test was performed for each strain using the percent values obtained by the “countcolors” program, comparing each strain with a control strain as described above.

### Stability testing of enterobactin.

To test the stability of enterobactin under conditions used in the liquid growth experiments, pure enterobactin was resuspended at 1 mM in H_2_O and diluted 1:2 to 500 μM in a 2× solution of 2× M9, 200 mM glucose, and 2 μM FeCl_3_, either with or without 16 μg/ml catalase. Twenty microliters of 500 μM enterobactin under each experimental condition (with or without catalase) was added to three replicate wells on a 384-well plate, starting at time zero. Every 2 h, another row of 20-μl triplicates was added, until 6 h, and incubated at 37°C in a Tecan Infinite M Nano spectrophotometer (Tecan Inc., Männedorf, Switzerland). At 6 h, fresh enterobactin 20-μl aliquots were added for the zero time point, and samples representing all time points were assayed using Arnow’s assay by adding 2 μl 5 N HCl and 10 μl Arnow’s reagent, followed by 2 μl 10 N NaOH. Catecholate absorbance was measured with an Infinite M Nano spectrophotometer (Tecan Inc.) at 500 nm.

### Calmagite testing of pure enterobactin.

Enterobactin is best known for its ability to bind the insoluble trivalent iron (Fe^3+^). In order to assess the ability of enterobactin purified from *R. mucilaginosa* to bind magnesium and zinc, the compleximetric dye calmagite was used in an assay format (41, 42). Calmagite forms colored complexes with magnesium and zinc as well as other metals and was developed to quantify magnesium in biological samples (41). A chelating compound, such as EDTA or enterobactin, is able to break this complex and elicit a color change from red to blue that can be measured spectrophotometrically. This assay was adapted to the analysis of enterobactin as follows: calmagite dye (Acros Organics) was diluted in DI H_2_O to 0.4 mg/ml. A working solution was prepared consisting of a 25 μM metal ion solution (either MgSO_4_ or ZnSO_4_ diluted in DI H_2_O) and 0.05-mg/ml calmagite compleximetric dye diluted in 62.5 mM NH_4_Cl, pH 10. One hundred seventy-five microliters was mixed with either 25 μl of a dilution series of EDTA (for quantification via a standard curve) or 25 μl of enterobactin sample at 896 μM and 100 μM (unknowns). The standard curve and unknown samples were all assayed in triplicate, and the disappearance of the red calmagite-metal complex due to EDTA or siderophore metal binding competition was measured at 650 nm in a Tecan Infinite M Nano spectrophotometer set at room temperature. EDTA equivalents of enterobactin were calculated by extrapolating enterobactin absorbance values from third-order polynomial line-fitting of standard curve data (see Fig. S7 in the supplemental material).
