# Supplementary figures and images for: Commensal Oral Rothia mucilaginosa Produces Enterobactin, a Metal-Chelating Siderophore
Source: mSystems. 2020 Apr 28;5(2):e00161-20. doi: 10.1128/mSystems.00161-20 (PMC7190385; doi:10.1128/mSystems.00161-20)

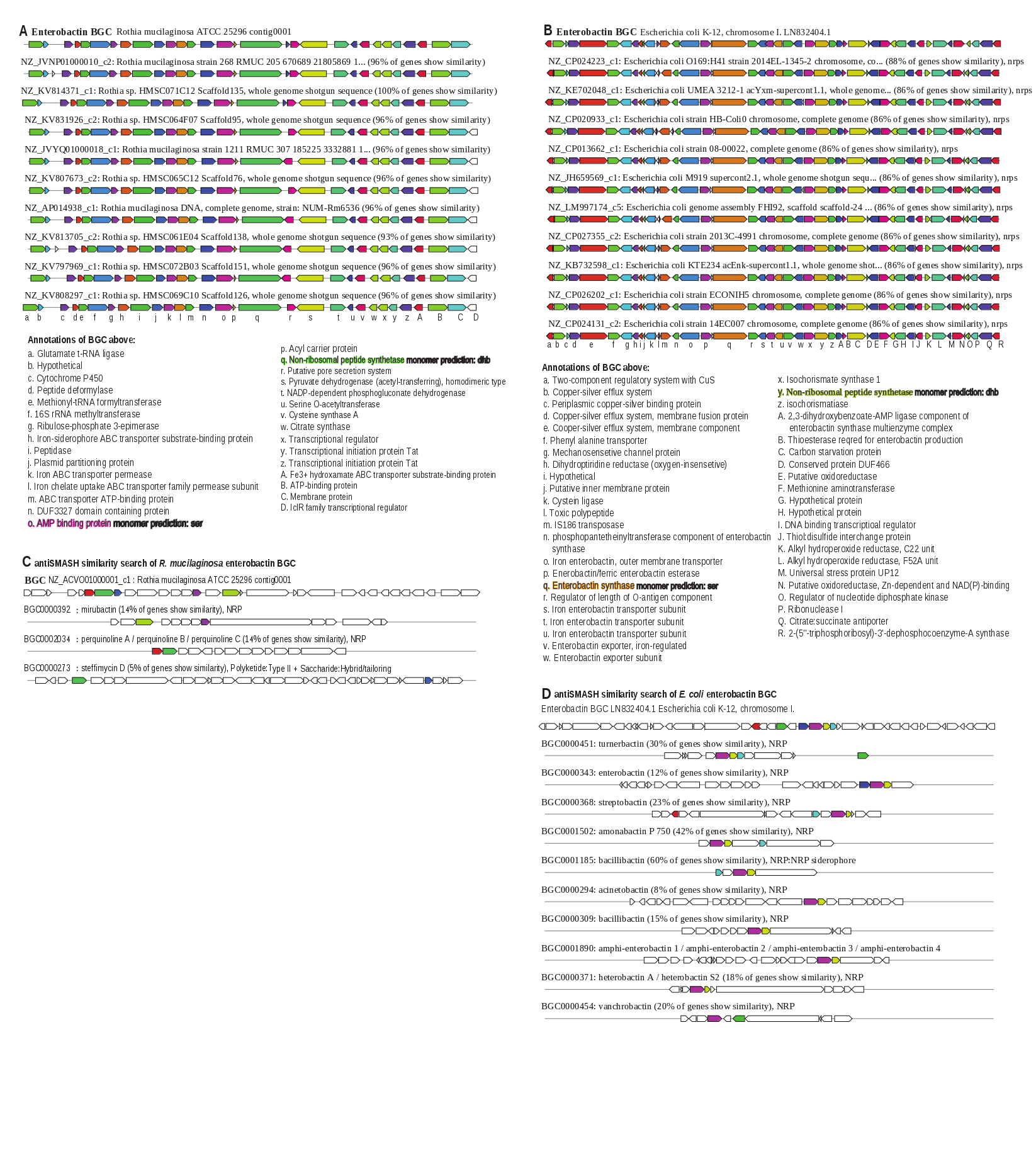

Supplement: FIG S1 [file mSystems.00161-20-sf001.tif]

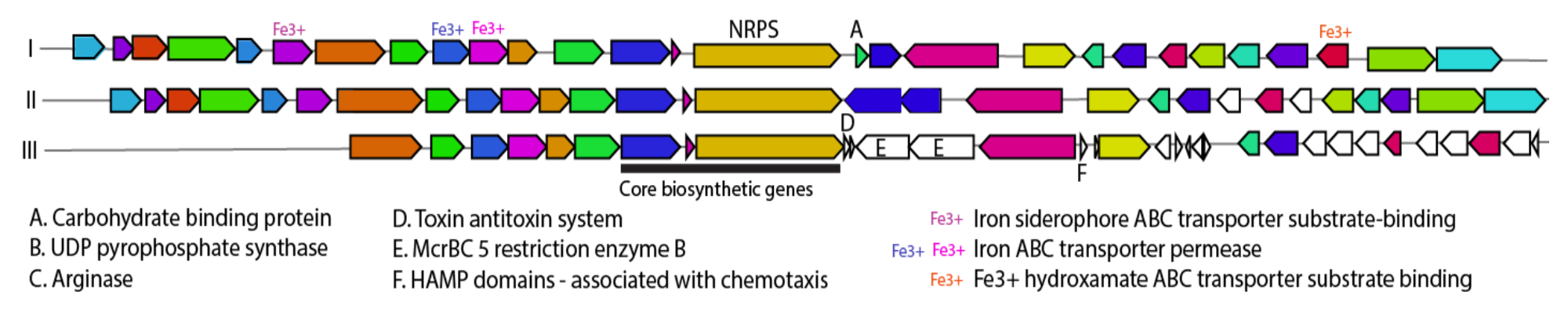

Supplement: FIG S2 [file mSystems.00161-20-sf002.tif]

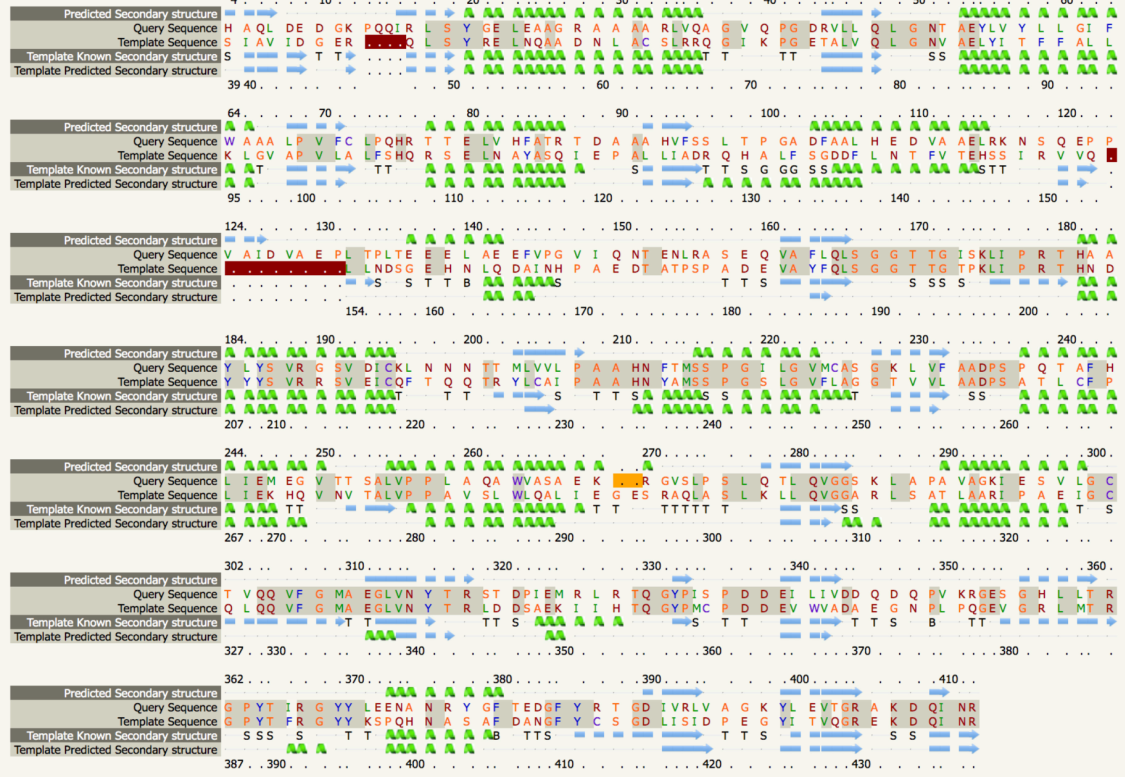

Supplement: FIG S3 [file mSystems.00161-20-sf003.tif]

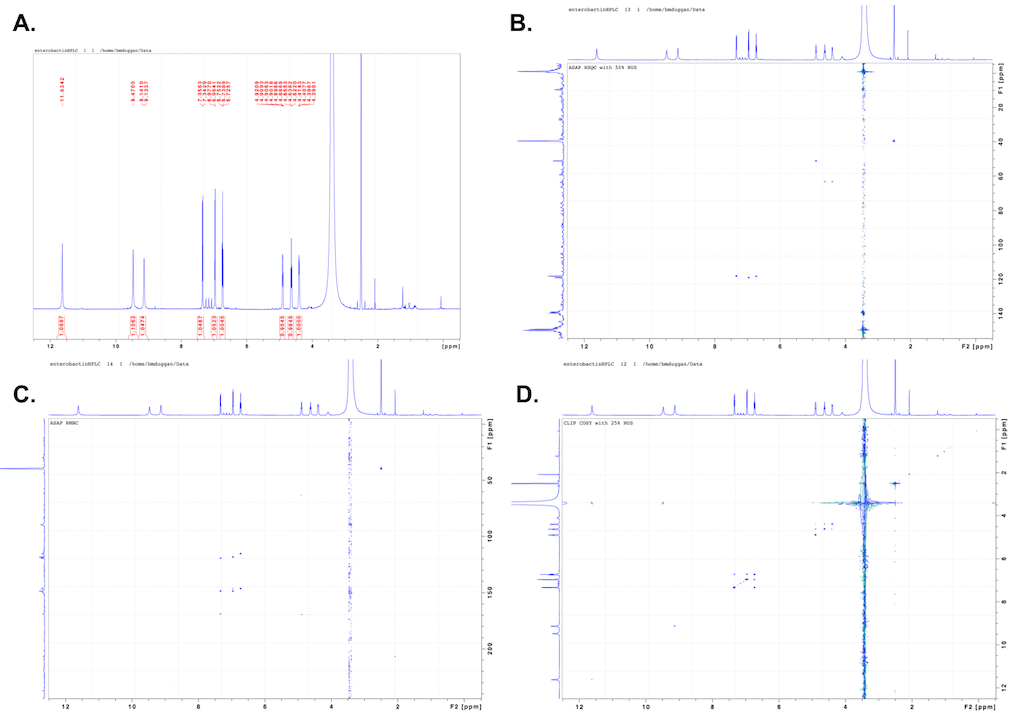

Supplement: FIG S4 [file mSystems.00161-20-sf004.tif]

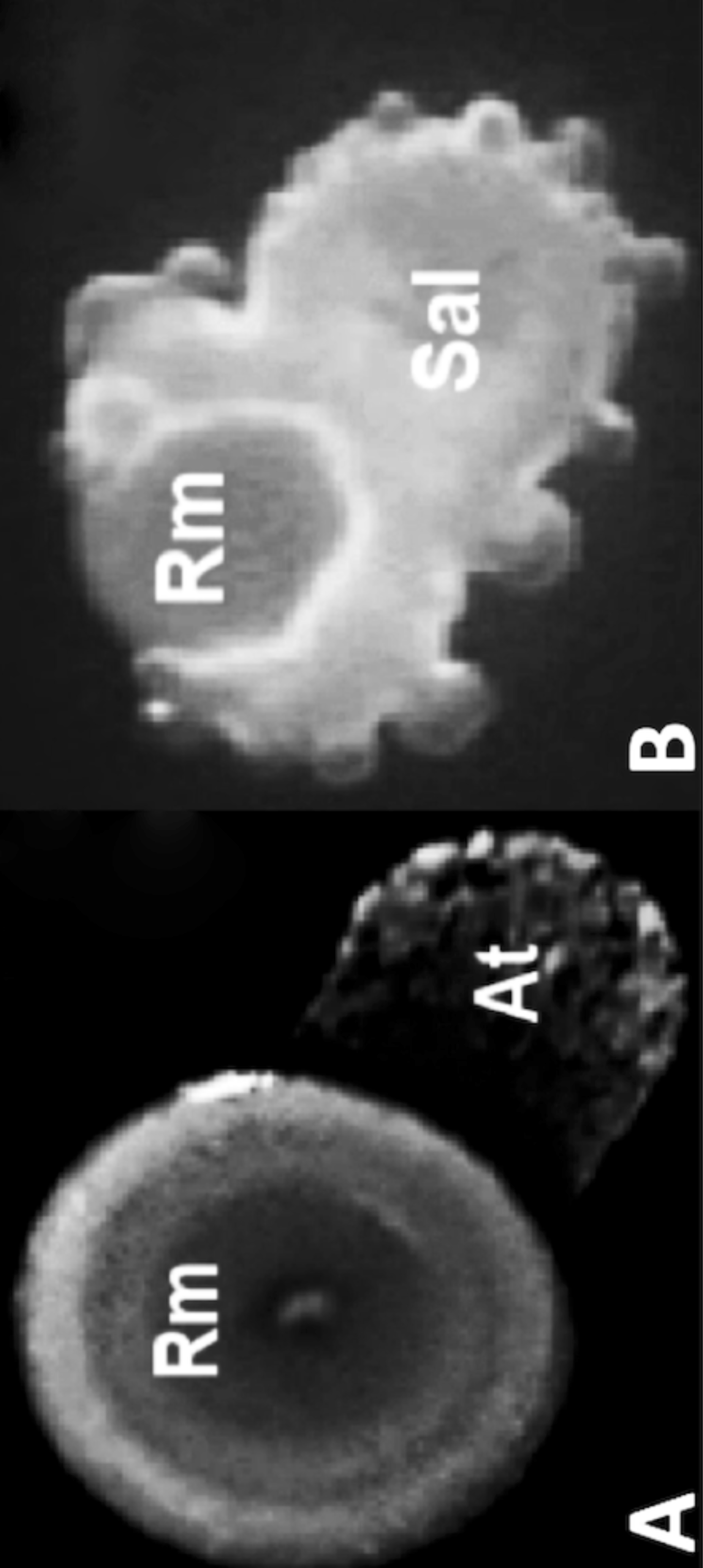

Supplement: FIG S5 [file mSystems.00161-20-sf005.tiff]

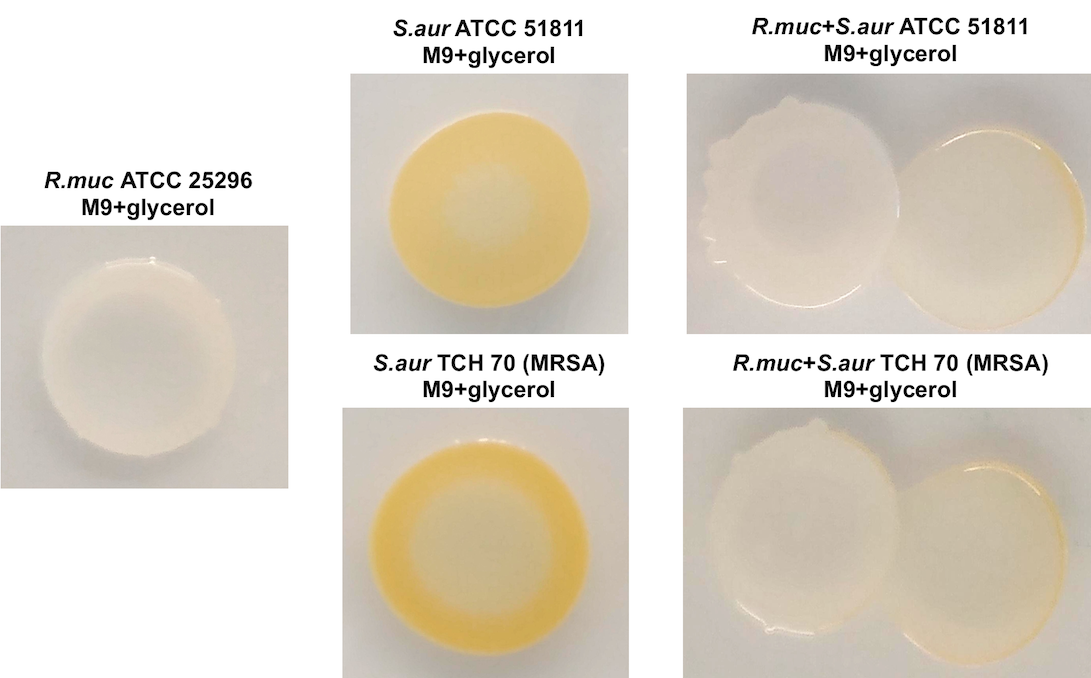

Supplement: FIG S6 [file mSystems.00161-20-sf006.tif]

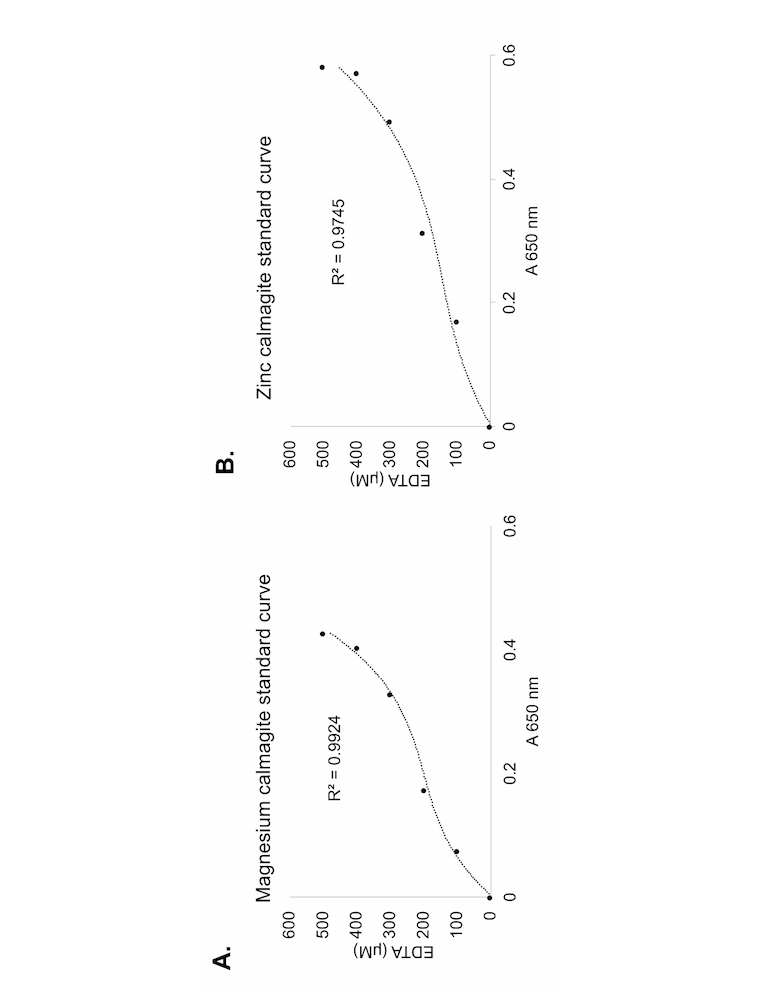

Supplement: FIG S7 [file mSystems.00161-20-sf007.tif]
