# Supplementary material for: Commensal Oral Rothia mucilaginosa Produces Enterobactin, a Metal-Chelating Siderophore
Source: mSystems. 2020 Apr 28;5(2):e00161-20. doi: 10.1128/mSystems.00161-20 (PMC7190385; doi:10.1128/mSystems.00161-20)
Supplement: TABLE S1 [file mSystems.00161-20-st001.pdf]

Table S1. Experimental ^{13}C and ^{1}H chemical shifts (ppm) of enterobactin produced by *Rothia mucilaginosa* ATCC 25296 in DMSO-d_6. All chemical shifts in this work are identical to the already characterized enterobactin compound (37).

Atom #	^{13}C (ppm)	Type	^{1}H (ppm)	Multiplicity	J (Hz)
Solvent: DMSO-d6					
1	169.0	C	-	-	-
2	51.1	CH	4.90	m	-
3	63.2	CH_2	4.63	t	9.8
			4.40	dd	11.1,4.1
4	168.5	C	-	-	-
5	114.7	C	-	-	-
6	148.1	C	-	-	-
7	145.7	C	-	-	-
8	119.1	CH	6.96	d	8.2
9	118.3	CH	6.73	t	7.9
10	118.1	CH	7.34	d	8.2
NH	-	-	9.13	br	-
6-OH	-	-	11.62	br	-
7-OH	-	-	9.47	br	-

“m”=multiplet, “t”=triplet, “d”=doublet, “dd”=doublet of doublets, “br”=broad “-“= not applicable.
